# The Crosstalk between Calcium Ions and Aldosterone Contributes to Inflammation, Apoptosis, and Calcification of VSMC via the AIF-1/NF-*κ*B Pathway in Uremia

**DOI:** 10.1155/2020/3431597

**Published:** 2020-12-04

**Authors:** Jianbing Hao, Jie Tang, Lei Zhang, Xin Li, Lirong Hao

**Affiliations:** ^1^Department of Nephropathy and Hemodialysis, First Affiliated Hospital of Harbin Medical University, Harbin, China; ^2^Department of Nephropathy, Southern University of Science and Technology Hospital, Shenzhen, China

## Abstract

Vascular calcification is a major complication of maintenance hemodialysis patients. Studies have confirmed that calcification mainly occurs in the vascular smooth muscle cells (VSMC) of the vascular media. However, the exact pathogenesis of VSMC calcification is still unknown. This study shows that the crosstalk between calcium and aldosterone via the allograft inflammatory factor 1 (AIF-1) pathway contributes to calcium homeostasis and VSMC calcification, which is a novel mechanism of vascular calcification in uremia. *In vivo* results showed that the level of aldosterone and inflammatory factors increased in calcified arteries, whereas no significant changes were observed in peripheral blood. However, the expression of inflammatory factors markedly increased in the peripheral blood of uremic rats without aortic calcification and gradually returned to normal levels with aggravation of aortic calcification. *In vitro* results showed that there was an interaction between calcium ions and aldosterone in macrophages or VSMC. Calcium induced aldosterone synthesis, and in turn, aldosterone also triggered intracellular calcium content upregulation in macrophages or VSMC. Furthermore, activated macrophages induced inflammation, apoptosis, and calcification of VSMC. Activated VSMC also imparted a similar effect on untreated VSMC. Finally, AIF-1 enhanced aldosterone- or calcium-induced VSMC calcification, and NF-*κ*B inhibitors inhibited the effect of AIF-1 on VSMC. These *in vivo* and *in vitro* results suggest that the crosstalk between calcium ions and aldosterone plays an important role in VSMC calcification in uremia via the AIF-1/NF-*κ*B pathway. Local calcified VSMC induced the same pathological process in surrounding VSMC, thereby contributing to calcium homeostasis and accelerating vascular calcification.

## 1. Introduction

Cardiovascular disease caused by vascular calcification is the main complication of chronic kidney disease (CKD), particularly in maintenance hemodialysis (MHD) patients [1–3]. Increasing evidence suggests that aldosterone plays an essential role in vascular calcification, and an aldosterone receptor antagonist alleviates vascular calcification [4]. However, due to side effects such as hyperkalemia, the use of aldosterone receptor antagonists in CKD is limited. Therefore, elucidating the mechanism of aldosterone in vascular calcification will provide a theoretical basis for the development of new practical solutions.

Aldosterone is involved in the inflammation of various tissues [5, 6]. Our previous study also successively confirmed that aldosterone contributes to inflammation of different tissues involved in CKD such as the heart, kidney, and peritoneum [7]. This suggests that aldosterone is a significant cause of chronic inflammation. Studies have suggested that the inflammatory response and macrophage activation are downstream targets of aldosterone. Aldosterone activates human macrophages and induces an inflammatory response [8]. However, aldosterone upregulates the expression of inflammatory factors (IL-16 and CTLA4) in human VSMC via an MR-dependent pathway [9, 10]. Aldosterone receptor antagonists alleviate the inflammatory response [11] and dysfunction of VECs [12]. However, aldosterone also exerts proinflammatory effects on VSMC via the MR-independent pathway, which is a rapid and nongenomic response [9]. In addition, the rapid and nongenomic effects of aldosterone are not blocked by MR antagonists [13, 14]. Under pathological conditions, aldosterone inhibits the expression of the calcium pump on the sarcoplasmic reticulum of VSMC [15], leading to intracellular calcium overload, cell damage, apoptosis, and calcification [16]. Christ and Wehling [17] proposed that aldosterone regulates calcium ion concentrations in lymphocytes, VECs, and VSMC. However, the effect of extracellular calcium ions on aldosterone synthesis in VSMC has not been investigated. Gao et al. [18] suggested that both intracellular and extracellular calcium ions could regulate aldosterone synthesis in the adrenal glands. This suggests that extracellular calcium can also affect aldosterone synthesis. Therefore, it is essential to confirm the interaction of aldosterone and calcium ions in VSMC.

Increasing evidence suggests that the process of vascular calcification is similar to the inflammatory response [19, 20]. There is prevalent inflammation and calcification in heart valves and aorta of MHD patients. However, the relationship between inflammation and ectopic calcification (vascular calcification) remains unclear. Vascular calcification in CKD mainly occurs in the media of the artery and involves the interaction of macrophages, VECs, and VSMC. Activated macrophages adhere to VECs and migrate across the endothelium to participate in vascular calcification [21]. The expression of inflammatory factors such as IL-1 beta, IL-6, MCP-1, and THF-alpha significantly increased in a rat model of vascular calcification [22], and these directly or indirectly participate in vascular calcification [23]. New and Aikawa have previously defined vascular calcification as a chronic inflammatory disease [24].

Allograft inflammatory factor 1 (AIF-1) is an EF-hand domain and calcium-binding protein. In addition, the EF-hand domain has the ability to bind calcium ions. When the EF-hand binds calcium ions, it undergoes a conformational change, leading to AIF-1 activation [25, 26] and contributing to macrophage activation and vascular inflammation [27]. Sommerville et al. proposed that AIF-1 is an inflammation-responsive protein-activated VSMC in AIF-1 overexpression mice [28]. Upon binding to Ca^2+^, AIF-1 is activated and induces inflammation, apoptosis, oxidative stress, and other pathological processes. Under normal physiological conditions, AIF-1 is not expressed in VSMC but is rapidly upregulated after exogenous stimulation [29–31]. Overexpression of AIF-1 regulates cyclin, and cytoskeletal protein leads to cell migration and phenotypic transformation [32], resulting in vascular inflammatory response and atherosclerosis. AIF-1 mediates atherogenesis-initiated signaling and activation of macrophages [33]. Structurally, the encoding gene of AIF-1 is located in the major histocompatibility complex class III region of chromosome 6p21.3, which is also densely populated with various inflammatory response protein genes such as the *TNF-α*, *TNF-β*, and *NF-κB* genes [34, 35]. NF-*κ*B plays a vital role in the inflammatory response. It has been confirmed that the chronic inflammatory response mediated via NF-*κ*B accelerates vascular calcification [36] and is involved in the pathogenesis of VSMC calcification [37]. Thus, there may be an interaction between AIF-1 and NK-*κ*B in inflammation and calcification of VSMC.

Clinically, it has been observed that total calcium content is normal, high, or low in MHD patients with normal serum calcium concentrations [38, 39]. Therefore, serum calcium concentrations cannot reflect total calcium content [40]. The calcium balance is much more complex in patients with chronic renal failure. Previous studies have confirmed that in most situations, total calcium content is higher in MHD patients and is associated with renal calcium excretion, dialysate calcium concentration, secondary hyperparathyroidism, and vitamin D metabolites [41–43]. Positive calcium balance readily causes calcium salt deposition in soft tissues, particularly in the arteries. Consequently, vascular calcification also possibly contributes to calcium homeostasis in MHD patients.

Therefore, aldosterone, AIF-1/NK-*κ*B, and inflammation are involved in calcium balance and vascular calcification in MHD. However, the underlying mechanism remains unclear. Our present study revealed that there is crosstalk between calcium ions and aldosterone mediated by the AIF-1/NK-*κ*B pathway, thereby contributing to inflammation, apoptosis, and calcification of VSMC.

## 2. Materials and Methods

### 2.1. Patients

In accordance with the clinical study program approved by the ethics committee of Harbin Medical University and the ethical standards of the 1964 Helsinki Declaration, we randomly collected a 2-mm discarded radial artery from 40 MHD patients who received autologous arteriovenous fistula surgery for the first time and 20 patients without any underlying disease who underwent surgery due to emergency forearm trauma. Then, the collected vascular specimens were fixed in 4% paraformaldehyde, embedded in paraffin, and used in subsequent analyses. Simultaneously, discarded serum samples from all enrolled patients were collected and cryopreserved after routine testing. All of the patients signed an informed consent form allowing the use of surgically discarded blood vessels and serum specimens before surgery.

### 2.2. Abdominal Aortic Calcification Score

Abdominal aortic calcification (AAC) of all enrolled patients was evaluated by lateral abdominal X-ray photographs, which included 11–12 thoracic vertebrae, 1–5 lumbar vertebrae, and 1–2 sacral vertebrae as described in a previous study [44]. The abdominal artery is a tubular structure in front of the spine. The calcification of the abdominal aorta corresponding to the 1–4 lumbar spine was independently scored by two blinded radiologists, and the average of the two scores of each aorta was used as the final score. The scoring method based on the range of calcification was as follows: without calcification, 0 points; <1/3 of the artery length, 1 point; between 1/3 and 2/3 of the artery length, 2 points; and >2/3 of the artery length, 3 points. The total score of AAC in each patient ranged from 0 to 24 points. According to AAC score and the segmented method in the CORD study, AAC ≤4 was noncalcification or mild calcification, 5 ≤ AAC ≤ 15 was moderate calcification, and AAC ≥16 was severe calcification [45].

### 2.3. Animal Model

Price et al. previously suggested that a synthetic diet (2.5% protein diet containing 0.75%) adenine produces consistent and dramatic medial calcification in adult rats within just 4 weeks [46]. In addition, we also have earlier verified the feasibility of this method [47]. Therefore, the rat model of uremia with aortic calcification was established by feeding SD rats (eight-week-old SD rats, weight range: 150–200 g, males and females were equally divided) with calcification-induced diet (2.5% protein diet containing 0.75% adenine) for 2, 4, 8, and 12 weeks as described in previous studies [46, 47]. The rats were divided into two groups as follows: the model group was fed with calcification-induced diet only; treatment group patients were given eplerenone (50 mg/kg·d, Pfizer), which is a selective mineralocorticoid receptor antagonist. At the end of the experiment, the rats were anesthetized (pentobarbital sodium, 150 mg/kg) by intraperitoneal injection and euthanized. The thoracic aortas and blood specimens were collected from each rat as soon as possible [47]. Whole animal experiments were conducted according to research protocols approved by the Animal Ethics Review Committee of Harbin Medical University and adhered to the principles stated in the Guide for the Care and Use of Laboratory Animals published by the US National Institutes of Health. Untreated rats were used as controls.

### 2.4. Cell Culture

Rat macrophages (Sciencell Research Laboratories, San Diego, California, USA) and VSMC (ATCC, USA) were cultured in a special medium for each cell line according to the manufacturer's instructions. When the cells reached 80% confluency, the cell culture medium was replaced with serum-free medium to cell synchronization for 24 h. Then, the cells were exposed to a high concentration of calcium ions (CaCl_2_, 1.5 mmol/L) or aldosterone (100 nM, Sigma, USA) for 12 h, 24 h, and 48 h. The untreated cells were cultured with the supernatant of cells treated with aldosterone or calcium ions. The cells and supernatant were harvested and analyzed. Calcification of VSMC was evaluated using the Red S staining assay (Sciencell Research Laboratories, USA).

### 2.5. Plasmid Constructs and Transfections

The AIF-1 gene overexpression construct was generated as described in our previous study [47]. The AIF-1 coding sequence was amplified and subcloned into a pcDNA3.1 vector (Invitrogen), generating the AIF-1 overexpression vector (pcDNA3.1-AIF-1). Plasmid pcDNA3.1-AIF-1 was transfected into cells through the transfection agent (riboFECT™ CP, China). All of the constructed plasmids were sequenced for validation.

### 2.6. Western Blotting

The total proteins in tissues or cells were extracted using a total protein extraction kit and quantified with a BCA protein kit (Solarbio Life Sciences, China). Fifty micrograms of total protein was loaded onto 12% SDS-PAGE gels and isolated. Then, the isolated proteins in SDS-PAGE gels were transferred to a PVDF membrane (Millipore, Billerica, MA, USA). The membranes were incubated in blocking buffer for western blotting (Solarbio Life Sciences, China) in sealed bags at room temperature for 1 h and then with primary antibodies against AIF-1, NF-*κ*B p65, p-NF-*κ*B p65 (Ser536, dilution 1 : 1,000, Abcam, UK), CYP11B2, CCR-2, and *β*-actin (dilution 1 : 1,000, Santa Cruz, USA) at 4°C overnight. After the primary antibody was removed, the membranes were incubated by goat anti-rat or goat anti-rabbit antibody (dilution 1 : 10,000, DyLight®800, Immuno Reagents, USA) at room temperature for 1 h and washed with Tris-buffered saline Tween (TBST). The bands were visualized and analyzed by an Odyssey Imaging System (LICOR Bioscience, USA).

### 2.7. Histopathological Analysis of Calcium Deposits

The discarded radial arteries of humans or aorta of rats were fixed with 4% paraformaldehyde, embedded in paraffin, and sectioned (4 microns in thickness). Calcification of vascular tissue sections was assessed using a Von Kossa kit according to the manufacturer's instructions. However, VSMC in 6-well plates was fixed in 4% paraformaldehyde. Cell calcification was evaluated by alizarin red staining kit according to the manufacturer's instructions. Finally, staining was assessed, and photomicrographs were captured under a microscope (Nikon Corp., Japan).

### 2.8. Quantification of Calcium Content and ALP Activity

The aorta or VSMC was dissolved in HNO_3_, and calcium content was measured using tissue and cell colorimetric method kits (Genmed Scientifics, USA) as previously described [48]. Subsequently, the measurement of calcium ion was normalized according to the total protein. Moreover, the ALP activity of aortic tissue and VSMC was measured using an ALP colorimetric assay kit (Abcam, UK), according to the manufacturer's protocol.

### 2.9. Immunohistochemical Staining for Histological Analysis

Specimens of the discarded radial artery in humans were fixed with 10% buffered formalin and embedded in paraffin, and 4-micron tissue sections were prepared. Paraffin sections were dewaxed, and endogenous peroxidase activity was blocked using 3% hydrogen peroxide at room temperature for 10 min (dark). Antigens were repaired by microwave and blocked with normal goat or rabbit serum (homologous to the second antibody animal serum) 37°C for 15 min. Then, the tissue sections were incubated with primary antibodies against CYP11B2, MR, and AIF-1 (1 : 1,000, Abcam, UK) at 4°C overnight. After rinsing with PBS, the tissue sections were incubated with goat anti-rat or goat anti-rabbit antibody at 37°C for 1 h. Finally, the immunocomplexes were stained with DAB and observed under a microscope (Nikon Corp., Japan). Immunohistochemical staining was independently evaluated by two experienced, blinded pathologists. Based on relative immunostaining intensity, the results were classified into four categories as follows: “0” as negative, “+1” as mild, “+2” as moderate, and “+3” as strong. The final results were averaged.

### 2.10. Macrophage Phenotype (M1/M2) Analysis

M1 macrophages impart proinflammatory effects, whereas M2 macrophages exhibit anti-inflammatory effects. It has been suggested that the exchange of macrophage phenotype (M1/M2) is associated with chronic low-grade inflammation. Thus, the overall balance between M1 and M2 macrophages is associated with equilibrium state of inflammation. The ratio of M1/M2 macrophages may thus be utilized in predicting the level of inflammation. Briefly, 400 *μ*L of the blood sample was taken and centrifuged at 4°C, and the pellet containing the erythrocytes was collected and fixed with 4% formaldehyde. M1 macrophages were labeled with antibodies against CD68 and CCR2, and M2 macrophages were labeled with antibodies against CD163 and CX3CR1. Subsequently, the ratio of M1/M2 macrophages in the blood was detected and calculated by flow cytometry.

### 2.11. Apoptosis Assay

Experimental cells were harvested and washed twice with PBS and assayed by an Annexin V-FITC/PI apoptosis detection kit (Beijing Solarbio Science & Technology Co., Ltd., China). Briefly, 300 *μ*L of binding buffer was added to resuspend the cells, and 5 *μ*L of Annexin V-FITC was added. After mixing, the cells were incubated at room temperature for 15 min. Five microliters of PI was added 5 min before detection. Finally, 200 *μ*L of 1× binding buffer was added. The rate of cell apoptosis was analyzed by flow cytometry (BD Accuri C6, USA). The same experiment was repeated thrice in parallel.

### 2.12. ELISA

The levels of aldosterone, hypersensitive C-reactive protein (hs-CRP), MCP-1, and AIF-1 in serum or supernatant were detected using an ELISA kit, according to the manufacturer's instructions.

### 2.13. Statistical Analysis

The SPSS 19.0 software was used for statistical analysis of all of the experimental data. The measurement data of normal distribution were presented as the mean ± standard deviation, and the nonnormal distribution data were expressed as the median and quartile spacing. One-way ANOVA or Wilcoxon rank-sum test was used for comparison among groups, and the chi-square test was used for enumerating the data. Differences with *P* < 0.05 were considered statistically significant.

## 3. Results and Discussion

### 3.1. Clinicopathological Factors in the Serum of MHD Patients

Because calcium and phosphorus are the main raw materials of calcification, these play a key role in vascular calcification [49].  Table 1 shows that the levels of Ca^2+^, ALP activity, macrophage polarization (M1/M2), hs-CRP, aldosterone, AIF-1, MCP-1, and albumin in serum did not significantly change except for phosphate in MHD patients with arterial calcification. This indicated that there were no obvious abnormal indicators in the peripheral blood of MHD patients, which is discordant to the findings of an earlier study [50]. A possible explanation for this discrepancy is the degree of vascular calcification. We propose that the levels of calcium, aldosterone, and inflammation in peripheral blood and local vascular tissues vary.

It is well known that calcium homeostasis is regulated by intestinal calcium absorption, bone calcium remodeling, and renal calcium excretion. In particular, calcium metabolism is more complicated in patients with CKD or MHD [51, 52]. For example, in CKD stages 2–5, acidosis or renal tubular damage leads to a decrease in tubular calcium reabsorption, and 1,25(OH)_2_D synthesis decreases or hyperphosphatemia leads to a decrease in intestinal calcium absorption [53–55]. A decrease in tubular calcium reabsorption and intestinal calcium absorption results in hypocalcemia. However, MHD promotes a positive calcium balance and results in hypercalcemia for the following reasons: a decrease in glomerular filtration rate leads to a decrease in renal calcium excretion, progressive secondary hyperparathyroidism leads to an increase in bone resorption and tubular calcium reabsorption, the use of calcium-containing phosphate binders or vitamin D metabolites leads to an increase in intestinal calcium absorption, and calcium ions are present in the dialysate [56]. Bones are incapable of buffering excessive amounts of calcium [57] and thus are stored in soft tissue (ectopic calcification such as vascular calcification), which is beneficial to maintaining serum calcium levels. Consequently, vascular calcification may also contribute to calcium balance in MHD patients. It has been confirmed that calcium deposition onto vascular walls is an active process, but the exact mechanism remains unclear. Based on previous studies, we hypothesize that abnormal levels of calcium ions potentially activate macrophages to release inflammatory factors in MHD patients, which in turn leads to inflammation and calcification of VSMC.

### 3.2. Overproduction of Aldosterone and Inflammatory Factors in the Calcified Artery

**Figure 1:**
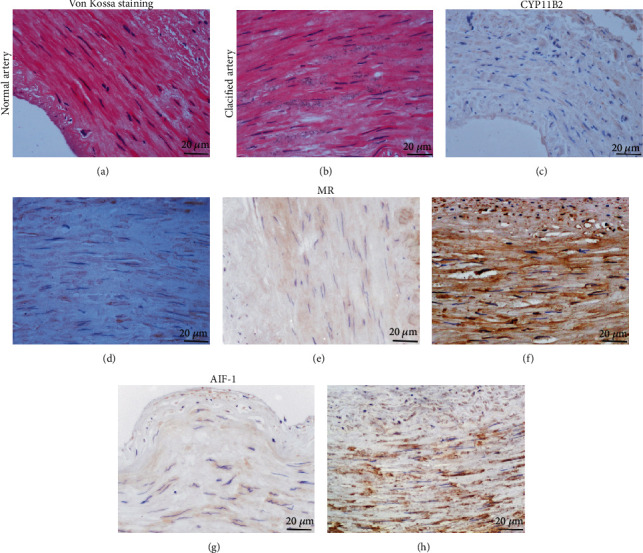
Histological analysis of radial artery in MHD patients. (a, b) Calcification in radial artery was evaluated by Von Kossa staining. (c–h) Expression of CYP11B2, MR, and AIF-1 in radial arteries was evaluated by immunohistochemical staining.

There is significant ectopic calcification such as the aorta, coronary artery, and heart valves in MHD patients [58, 59]. Vascular calcification is the main cause of cardiovascular accidents and loss of arteriovenous fistula. To investigate the pathogenesis of arterial calcification, we evaluated arterial calcification based on AAC scores and the histomorphology of the radial artery, which were abandoned during the operation of autologous arteriovenous internal fistula. Compared with a healthy individual, the AAC scores showed significant abdominal aortic calcification in 40 MHD patients. In the 40 MHD patients, 11 (27.5%) patients had mild calcification, 19 (47.5%) patients had moderate calcification, and 10 (25%) patients had severe calcification. In addition, the results of Von Kossa staining clearly showed the presence of calcium deposition in the radial artery of MHD patients (Figures 1(a) and 1(b)). Simultaneously, the aldosterone system (CYP11B2 and MR) and inflammatory factor (AIF-1) were significantly upregulated in the calcified radial artery and associated with the severity of calcification (Figures 1(c)–1(h), Table 2). Dysregulation of mineral metabolism promotes arterial calcification in uremic patients [60]. Our data from MHD patients with obvious arterial calcification showed that there was no obvious increase in the levels of blood calcium, aldosterone, and inflammatory factors that contributed to vascular calcification. It also indirectly indicated that vascular calcification occurs in local blood vessels. In addition, this study confirmed that aldosterone synthase and AIF-1 are overexpressed in the calcified radial artery of MHD patients with AAC ≥5.

**Figure 2:**
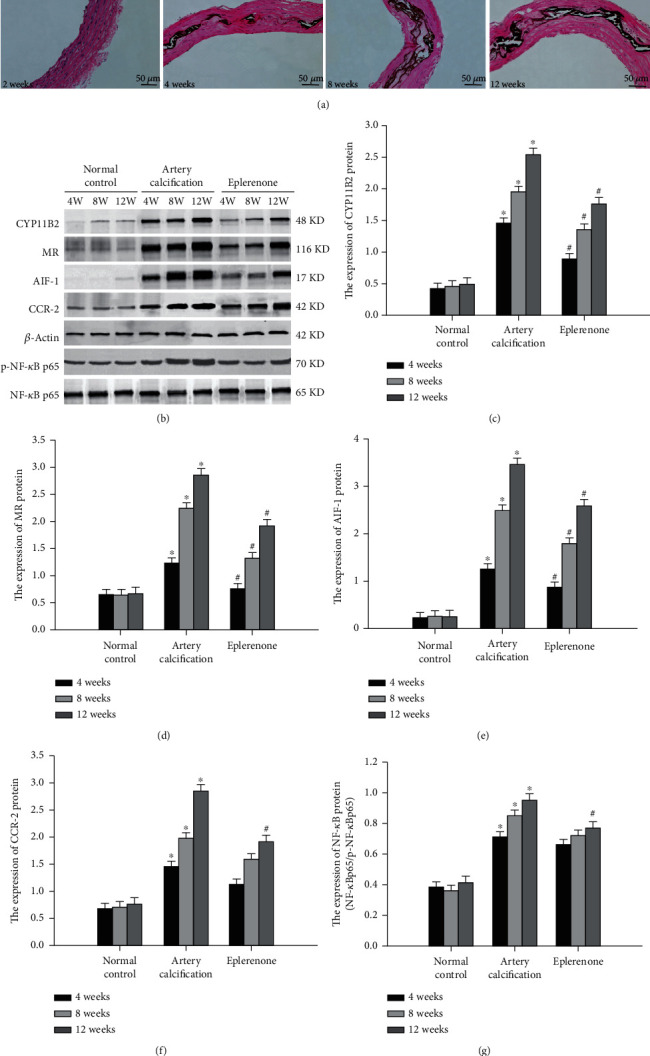
Aortic calcification and inflammatory factors in uremic rats. (a) Calcification in aortic artery was evaluated by Von Kossa staining. (b–f) Expression of CYP11B2, MR, AIF-1, and CCR-2 was evaluated by western blot assay (*n* = 8). (g) NF-*κ*B activity was evaluated by western blot assay (*n* = 8). Data were represented by means ± SD. Versus normal control group, ^∗^*P* < 0.01; versus artery calcification group, #*P* < 0.01.

There were similar changes in the uremic rat model with aortic calcification.  Figure 2(a) shows significant aortic calcification in uremic rats. Positive calcium balance likely causes calcium salt deposition in soft tissues, including the arterial walls, and, therefore, may be a strong predictor of the severity of vascular calcification [9, 47, 61]. Vascular calcification can be evaluated by calcium content. Meanwhile, *CYP11B2*, *MR*, *AIF-1*, *NF-κB*, and *CCR-2* in calcified aortas were significantly upregulated (Figures 2(b)–2(g)). Treatment with a mineralocorticoid receptor antagonist prevented and alleviated aortic calcification in the rat model. These findings suggest that the local overproduction of the aldosterone system (CYP11B2 and MR) and inflammatory factors (AIF-1, NF-*κ*B, MCP-1, and CCR-2) contributed to calcium metabolism and vascular calcification.

AIF-1 is an essential inflammatory factor that contributes to inflammation which is associated with various diseases [62]. Nevertheless, whether AIF-1 is also involved in vascular calcification remains unclear. As shown in the rat model results, AIF-1-related inflammatory factors in blood circulation increased before aortic calcification and decreased after aortic calcification. However, the expression of AIF-1-related inflammatory factors in the calcified aorta was significantly upregulated. This observation suggests that systemic inflammation affects the initiation of VSMC calcification, while local inflammation of the arterial wall contributed to the continuous progression of VSMC calcification. Elucidation of the pathogenesis of local inflammation in the artery may contribute to alleviating calcification of VSMC [63].

Maintaining calcium balance is a complex process in CKD. While hypocalcemia is common in nondialysis CKD patients, a positive calcium balance may be encountered in MHD patients [40, 51, 55, 64, 65]. As medial artery layers can accommodate large amounts of calcium, we propose that vascular calcification may contribute to calcium balance in MHD patients. However, the long-term persistence of arterial calcification will also lead to cardiovascular accidents. It has been suggested that local aldosterone and AIF-1 are involved in radial artery calcification. Studies have also demonstrated aldosterone-induced inflammatory responses in multiple cells, leading to fibrosis and calcification [66, 67]. However, the mechanism of inflammation induced by aldosterone and AIF-1 in VSMC requires further investigation.

### 3.3. Interaction of Calcium Ions and Aldosterone in Inflammation, Apoptosis, and Calcification of VSMC

**Figure 3:**
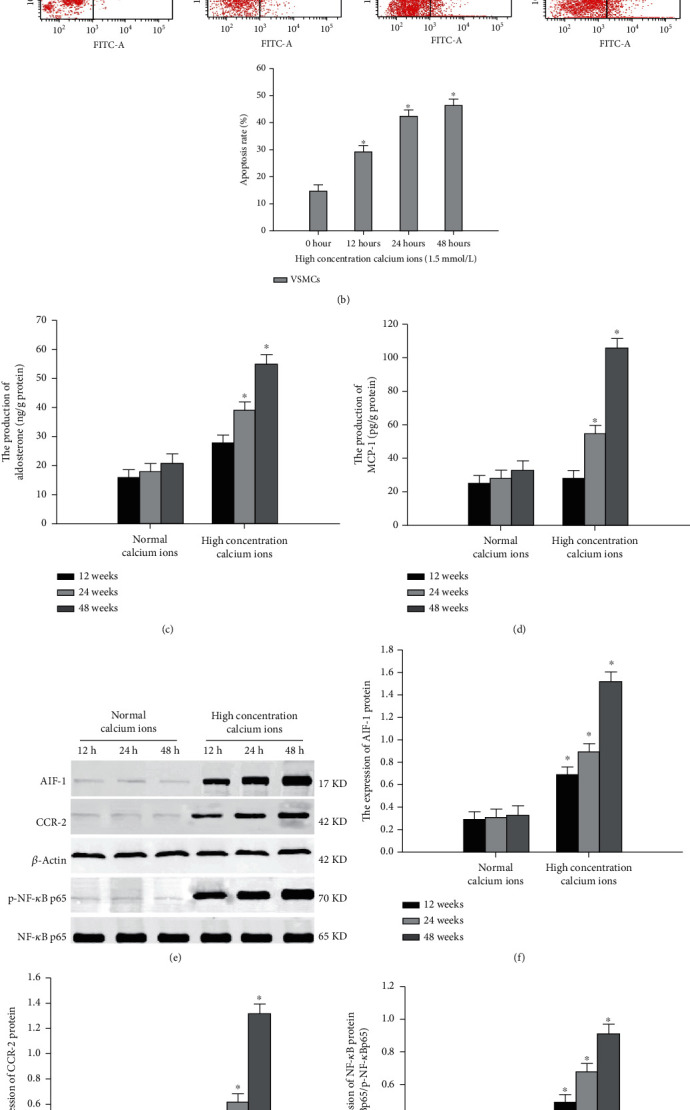
The effect of calcium ions on inflammation, apoptosis, and calcification in VSMCs. (a) Calcified nodules of VSMCs were detected by alizarin red staining. (b) Apoptosis of VSMCs was evaluated by flow cytometry. (c) Production of aldosterone in VSMCs was regulated by calcium ions. (d) Production of MCP-1 in VSMCs was regulated by calcium ions. (e–h) Expression of AIF-1, CCR-2, and NF-*κ*B in VSMCs was evaluated by western blot assay. Data were represented by means ± SD from three independent experiments. Versus normal calcium ions, ^∗^*P* < 0.01.

**Figure 4:**
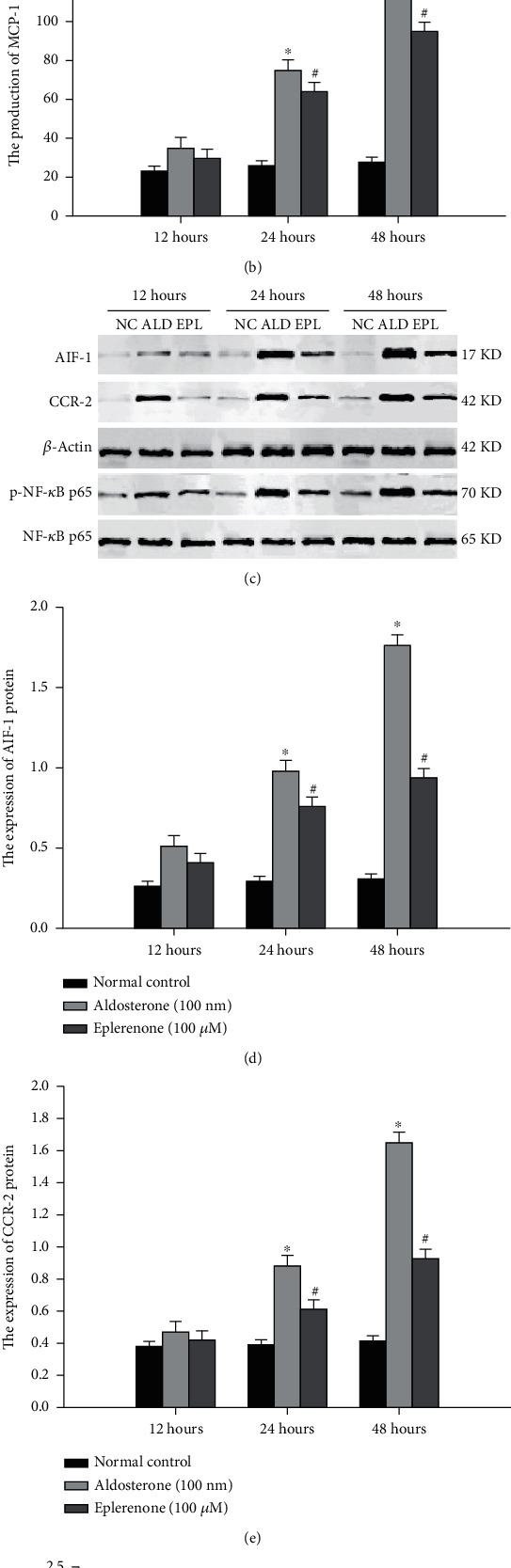
The effect of aldosterone on intracellular calcium ions and inflammation in VSMCs. (a) Intracellular calcium ions in VSMCs were affected by aldosterone. (b) Production of MCP-1 in VSMCs was regulated by aldosterone. (c–f) Expression of AIF-1, CCR-2, and NF-*κ*B in VSMCs was evaluated by western blot assay. Data were represented by means ± SD from three independent experiments. Versus normal control group, ^∗^*P* < 0.01; versus aldosterone group, #*P* < 0.01.

Although studies have confirmed that there is an interaction between calcium ions and aldosterone in many cells, its exact role in vascular calcification remains unclear. To clarify the role of calcium ions and aldosterone in inflammation, apoptosis, and calcification of VSMC, we cultured VSMC *in vitro* as follows. When VSMC were exposed to a high concentration of calcium ions (CaCl_2_, 1.5 mmol/L) for 0 h, 12 h, 24 h, and 48 h, compared with 0 h, calcified nodule formation and apoptosis of VSMC significantly increased after 24 h and 48 h (Figures 3(a) and 3(b)). In addition, the production of aldosterone, AIF-1, NF-*κ*B activity, MCP-1, and CCR-2 in calcified VSMC was upregulated (Figures 3(c)–3(h)). In turn, when VSMC were exposed to aldosterone (Aldo, 100 nM) for 12 h, 24 h, and 48 h (Figure 4), the levels of intracellular calcium ions, AIF-1, NF-*κ*B activity, MCP-1, and CCR-2 were enhanced at 24 h and 48 h compared with 12 h (Figures 4(a)–4(f)). In contrast, treatment with a mineralocorticoid receptor antagonist inhibited inflammation, apoptosis, and calcification of VSMC. These results suggest that the interaction between calcium ions and aldosterone contributed to inflammation, apoptosis, and calcification of VSMC. The AIF-1/NF-*κ*B/MCP-1/CCR-2-pathway might mediate the crosstalk between calcium ions and aldosterone.

### 3.4. Activated Macrophages Induce Initiation of VSMC Inflammation and Calcification

Previous *in vitro* and animal studies have confirmed that chronic inflammation induced by aldosterone is involved in vascular calcification in uremia. Clinical research has confirmed that significant arterial calcification occurs in MHD patients. Still, there was no obvious exchange of the macrophage phenotype (M1/M2), inflammatory factors, and aldosterone levels in peripheral blood. Previous studies have confirmed that macrophage polarization (M1 and M2) plays an important role in inflammation [68, 69]. The ratio of M1/M2 contributes to predict chronic inflammation.  Table 1 shows that macrophages (M1/M2) and inflammatory factors in the blood circulation of uremic patients with evident vascular calcification had no increase. However, these significantly increased in the early stage of uremic rats without arterial calcification and gradually returned to normal levels as artery calcification occurred and progressed (Table 3). However, the expression of aldosterone and inflammatory factors in the calcified artery was upregulated. These results indicate that aldosterone and inflammation in the artery play essential roles in the progression of vascular calcification. Our group hypothesizes that systemic inflammation is not the main cause of arterial calcification, and local inflammation possibly promotes the progress of vascular calcification. Dysregulation of calcium-activated macrophages leading to the release of inflammatory factors and aldosterone induces intracellular calcium ions overload, cell damage, inflammation, apoptosis, and calcification. Activated VSMC would synthesize and secrete aldosterone and inflammatory factors to amplify the inflammatory signal of macrophages and induce calcium ion storage in VSMC.

**Figure 5:**
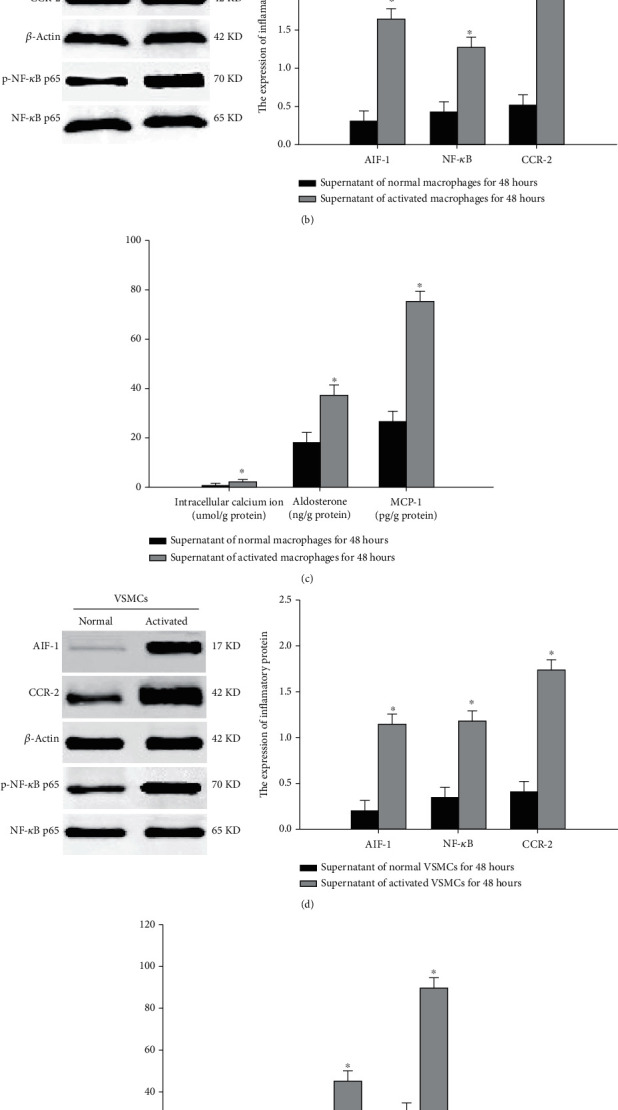
The effect of activated macrophages on inflammation and calcification in VSMCs. (a) Calcified nodules of VSMCs were detected by alizarin red staining. (b) Expression of AIF-1, CCR-2, and NF-*κ*B in VSMCs was affected by activated macrophages. (c) Intracellular calcium ions, aldosterone, and MCP-1 in VSMCs was affected by activated macrophages. (d) The effect of activated VSMCs on the expression of AIF-1, CCR-2, and NF-*κ*B in untreated VSMCs. (e) The effect of activated VSMCs on intracellular calcium ions, aldosterone, and MCP-1 in untreated VSMCs. Data were represented by means ± SD from three independent experiments. Versus normal group, ^∗^*P* < 0.01.

To explore the role of macrophages and VSMC in vascular calcification, untreated VSMC was cultured with the supernatant of macrophages, which were stimulated by a high concentration of calcium ions.  Figure 5 shows that the supernatant of activated macrophages promotes calcified nodule formation, induces intracellular calcium ion overloading and upregulates the expression of aldosterone, AIF-1, NF-*κ*B, MCP-1, and CCR-2 in VSMC (Figures 5(a)–5(c)). Untreated VSMC were cultured with the supernatant of VSMC that were exposed to the supernatant of activated macrophages. The supernatant of activated VSMC also promoted calcified nodule formation, activated aldosterone, and induced the expression of inflammatory factors in VSMC (Figures 5(a), 5(d), and 5(e)). These results indicated that activated VSMC possess abilities similar to activated macrophages on untreated VSMC. However, there was no significant change in aldosterone and inflammatory factors in macrophages that were exposed to the supernatant of activated VSMC. This indicated that VSMC had no direct effect on the activity of macrophages. These results suggest that macrophages activated by a high concentration of calcium ions induce inflammation, apoptosis, and calcification of VSMC via the aldosterone/AIF−1/NF-*κ*B pathway. Furthermore, activated VSMC play a similar role to the surrounding normal VSMC but did not directly influence the activity of macrophages. Thus, a vicious and self-sustained cycle is established, resulting in the continuous progression of VSMC calcification that is no longer dependent on macrophages. These dynamics could explain why the level of inflammatory factors in blood did not change with severe vascular calcification.

### 3.5. AIF-1 Mediates Aldosterone-Induced VSMC Inflammation, Apoptosis, and Calcification via the NF-*κ*B Pathway

**Figure 6:**
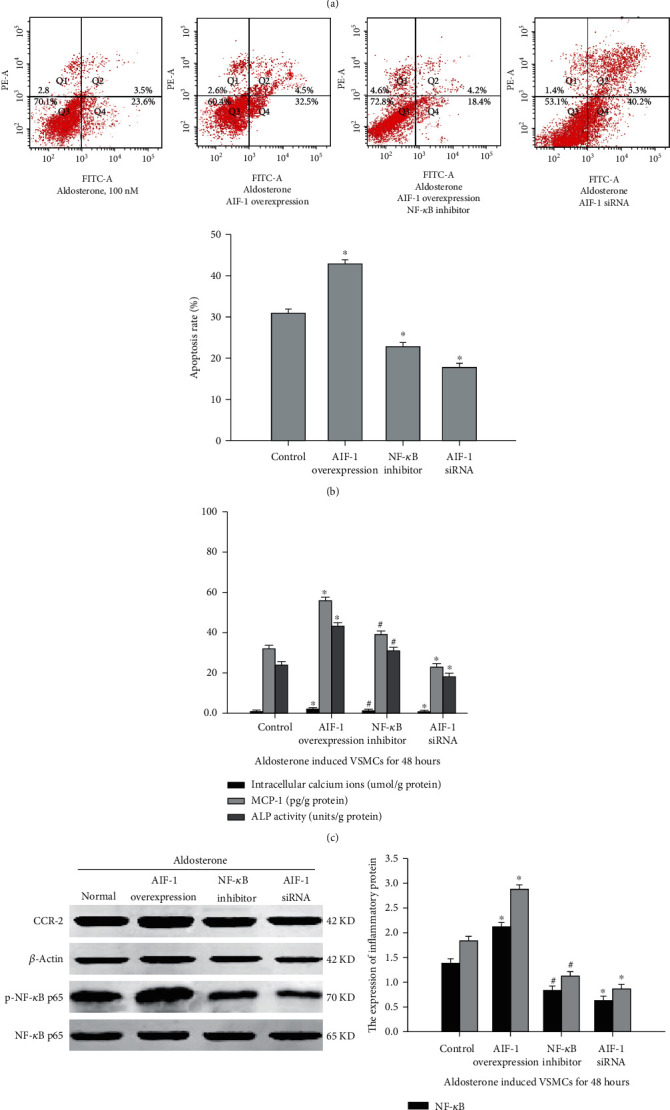
The effect of AIF-1/NF-*κ*B on inflammation, apoptosis, and calcification in VSMCs induced by aldosterone. (a) Calcified nodules of VSMCs were detected by alizarin red staining. (b) Apoptosis of VSMCs was evaluated by flow cytometry. (c) The effect of AIF-1/NF-*κ*B on intracellular calcium ions, MCP-1, and ALP activity in VSMCs exposed to aldosterone. (d) The effect of AIF-1/NF-*κ*B on inflammatory factors in VSMCs exposed to aldosterone. Data were represented by means ± SD from three independent experiments. Versus control group, ^∗^*P* < 0.01; versus AIF-1 overexpression group, #*P* < 0.01.

The gene that encodes AIF-1 is surrounded by various inflammatory genes, including *NF-κB*. AIF-1 regulates the inflammatory response by activating the surrounding inflammatory genes. It has been confirmed that the NF-*κ*B signal plays an essential role in vascular calcification. In addition, our previous study proved that AIF-1 contributes to the macrophage activity by acting as an inflammatory factor. Consequently, we hypothesize that AIF-1 participates in the process of vascular calcification via the NF-*κ*B pathway. To determine the role of AIF-1/NF-*κ*B in inflammation, apoptosis, and calcification of VSMC induced by aldosterone, AIF-1-overexpressing VSMC exposed to aldosterone for 48 h, which resulted in enhancement of the levels of calcified nodules, apoptosis, intracellular calcium ions, ALP activity, NF-*κ*B activity, and inflammatory factors (MCP-1/CCR-2) (Figure 6). Moreover, inhibition of NF-*κ*B activity reduced inflammation and calcification that was induced by aldosterone in VSMC AIF-1-overexpressing VSMC. When the expression of AIF-1 was inhibited in VSMC by exposure to aldosterone, the activity of NF-*κ*B was downregulated, inflammatory factors (MCP-1/CCR-2) and apoptosis were decreased, and calcification (calcified nodules, intracellular calcium ions, and ALP activity) was alleviated (Figure 6). These results suggest that AIF-1 contributes to aldosterone-induced inflammation, apoptosis, and calcification of VSMC by regulating NF-*κ*B activity. Through this mechanism, the overload of calcium ions continuously deposited in the middle membrane of the artery results in arterial calcification and maintains calcium homeostasis.

## 4. Conclusion

For the first time, we have demonstrated that the crosstalk between calcium ions and aldosterone contributes to the inflammation, apoptosis, and calcification of VSMC via the AIF-1/NF-*κ*B pathway in uremia. In addition, local calcified VSMC promote similar pathological processes in the surrounding VSMC, thereby resulting in vascular calcification. This study strongly suggests that the inhibition of the AIF-1/NF-*κ*B pathway is helpful to prevent inflammation, apoptosis, and calcification of VSMC.

## Figures and Tables

**Table 1:** Clinicopathological factors in serum.

Clinical parameters (median, IQR)	Normal patients (*n* = 50)	MHD patients (*n* = 100)	*P*
Ca^2+^ (mmol/L)	1.21 (1.14, 1.27)	1.33 (1.81, 1.41)	0.567
Phosphate (mmol/L)	1.48 (1.43, 1.65)	1.88 (1.51, 2.05)	0.034
Aldosterone (pg/mL)	90.6 (65.4, 116.5)	104.6 (71.4, 121.5)	0.085
ALP (unit/L)	95.5 (56.7, 126.5)	114.3 (75.6, 136.2)	0.066
Macrophage (M1/M2)	2.34 (1.81, 3.87)	3.18 (2.01, 4.37)	0.059
hs-CRP (mg/L)	5.34 (2.95, 7.59)	6.61 (3.58, 8.14)	0.831
AIF-1 (pg/mL)	152.5 (95.1, 186.5)	170.9 (127.1, 216.2)	0.052
MCP-1 (pg/mL)	135.8 (114.6, 166.2)	150.6 (125.1, 176.8)	0.064
Albumin (g/L)	42.1 (37.8, 44.7)	40.3 (36.2, 45.6)	0.728

**Table 2:** Expression of CYP11B2, MR, and AIF-1 in radial artery.

Variables	Noncalcification (%)	Calcification (%)			*P*
	0	+1	+2	+3	
CYP11B2					<0.001
0	0 (0.0)	0 (0.0)	0 (0.0)	0 (0.0)	
+1	20 (33.3)	1 (1.7)	0 (0.0)	0 (0.0)	
+2	0 (0.0)	2 (3.3)	2 (3.3)	1 (1.7)	
+3	0 (0.0)	0 (0.0)	10 (16.7)	24 (40.0)	
MR					<0.001
0	0 (0.0)	0 (0.0)	0 (0.0)	0 (0.0)	
+1	20 (33.3)	0 (0.0)	0 (0.0)	0 (0.0)	
+2	0 (0.0)	3 (5.0)	5 (8.4)	0 (0.0)	
+3	0 (0.0)	0 (0.0)	12 (20.0)	20 (33.3)	
AIF-1					<0.001
0	20 (33.3)	0 (0.0)	0 (0.0)	0 (0.0)	
+1	0 (0.0)	1 (1.7)	0 (0.0)	0 (0.0)	
+2	0 (0.0)	1 (1.7)	4 (6.7)	2 (3.3)	
+3	0 (0.0)	0 (0.0)	14 (23.3)	18 (30.0)	

Versus noncalcification, *P* < 0.001.

**Table 3:** Pathological factors in serum of uremic rats with arterial calcification.

Parameters (median, IQR)	2 weeks	4 weeks	8 weeks	12 weeks
Ca^2+^ (mmol/L)	1.41 ± 0.11	1.27 ± 0.09^∗^	1.38 ± 0.15	1.39 ± 0.16
Phosphate (mmol/L)	1.71 ± 0.18^∗^	1.98 ± 0.20^∗^	2.41 ± 0.21	2.55 ± 0.24^∗^
Aldosterone (pg/mL)	98.6 ± 8.22^∗^	90.6 ± 7.17^∗^	83.4 ± 7.01	73.5 ± 5.69
ALP (unit/L)	55.5 ± 4.13^∗^	64.3 ± 4.78	75.6 ± 5.78	86.2 ± 7.26^∗^
Macrophage (M1/M2)	10.3 ± 1.86^∗^	8.15 ± 1.16^∗^	5.01 ± 0.99	3.37 ± 0.45^∗^
hs-CRP (mg/L)	35.3 ± 3.27^∗^	16.6 ± 2.71^∗^	10.5 ± 1.84	5.14 ± 0.96^∗^
AIF-1 (pg/mL)	96.5 ± 7.67^∗^	81.9 ± 6.55^∗^	48.1 ± 4.78	46.2 ± 4.57
MCP-1 (pg/mL)	85.8 ± 8.42^∗^	88.6 ± 8.67^∗^	59.1 ± 5.67	56.8 ± 5.15

Versus 8 weeks, ^∗^*P* < 0.01. *n* = 8.

## Data Availability

All the data used to support the findings of this study are included within the article. If you have any request about our data, please feel free to use.
